# Impact of a digital health intervention on asthma resource utilization

**DOI:** 10.1186/s40413-018-0209-0

**Published:** 2018-12-03

**Authors:** Rajan Merchant, Stanley J. Szefler, Bruce G. Bender, Michael Tuffli, Meredith A. Barrett, Rahul Gondalia, Leanne Kaye, David Van Sickle, David A. Stempel

**Affiliations:** 1 Dignity Health, Woodland Clinic Medical Group, 632 W Gibson Rd, Woodland, CA 95695 USA; 2 Breathing Institute, University of Colorado School of Medicine and Children’s Hospital Colorado, 13123 East 16th Ave, Aurora, CO 80045 USA; 3 Division of Pediatric Behavioral Health, Department of Pediatrics, National Jewish Health, 1400 Jackson St, Denver, CO 80206 USA; 4 Propeller Health, 47 Maiden Lane, San Francisco, CA 94108 USA; 5 Propeller Health, 634 W Main Street #102, Madison, WI 53703 USA

**Keywords:** Telemedicine, Delivery of health care, Pulmonary medicine, Asthma, Digital health

## Abstract

Digital health interventions have been associated with reduced rescue inhaler use and improved controller medication adherence. This quality improvement project assessed the benefit of these interventions on asthma-related healthcare utilizations, including hospitalizations, emergency department (ED) utilization and outpatient visits. The intervention consisted of electronic medication monitors (EMMs) that tracked rescue and controller inhaler medication use, and a digital health platform that presented medication use information and asthma control status to patients and providers. In 224 study patients, the number of asthma-related ED visits and combined ED and hospitalization events 365 days pre- to 365 days post-enrollment to the intervention significantly decreased from 11.6 to 5.4 visits (*p* < 0.05) and 13.4 to 5.8 events (*p* < 0.05) per 100 patient-years, respectively. This digital health intervention was successfully incorporated into routine clinical practice and was associated with lower rates of asthma-related hospitalizations and ED visits.

## Introduction

Treatment paradigms for asthma management have been established and accepted [1]. Implementation of the guideline recommendations, however, has been suboptimal due to inconsistent and inadequate assessment of adherence to treatments and reporting of disease control [2]. Additionally, guideline implementation may be hindered by the intermittent nature of asthma symptoms, patient concern about potential adverse effects of therapy, and medication cost [3]. Inadequate adherence may result in patients failing to achieve asthma control [2].

Studies have shown that digital health interventions improve asthma control, reduce use of short-acting beta-agonist (SABA) “rescue” medications, increase the number of days without SABA use and improve adherence to controller medications [4–6]. A pragmatic trial demonstrated a significant reduction in SABA use and an increase in SABA-free days when SABA was monitored with electronic medication monitors (EMMs), and patients and their health care providers (HCPs) received feedback about medication use [7]. This subsequent quality improvement project was implemented to assess the impact of the digital health intervention on healthcare utilization in an open, single-arm study.

## Methods

Patients were enrolled from September 2014 to November 2017 during routine asthma care in specialty and primary care clinics. Inclusion criteria included provider diagnosis of asthma, prescription for SABA, Spanish or English fluency, and absence of other pulmonary disease or significant co-morbidity. Patients were prescribed medications according to standard guideline practice. Those who enrolled in a prior EMM clinical trial at Dignity Health were excluded [7].

Patients were provided digital EMMs (Propeller Health, Madison, WI) that attached to both controller and SABA inhaled medication(s). EMMs recorded date, time and number of puffs taken. The EMMs are part of a Food and Drug Administration (FDA) 510(k) cleared digital health platform consisting of mobile applications, web-based dashboards, and communication channels such as text messaging and email.

Patients authorized their HCPs to view their reports through a web interface, enabling them to integrate real-time information on SABA use and controller adherence into clinical decision-making [7].

Healthcare utilization information was collected from Dignity Health claims data for hospitalizations, emergency department (ED) visits and outpatient visits. These events were identified as asthma-related when specific codes (ICD9 493.XX or ICD10 J45.XX) were present in the primary billing position. Rate differences and their 95% confidence intervals were estimated to assess the change in pre-post utilization for ED visits, hospitalizations, combined ED visits plus hospitalizations, and outpatient visits, and *p*-values were calculated using Wilcoxon signed rank tests. For patients prescribed both SABA and controller medications, the controller-to-total medication ratio, defined as the number of controller medication puffs recorded divided by the total number of puffs of SABA plus controller medications, was calculated [8]. Patients were observed for 365 data days pre- and post-enrollment.

## Results

**Fig. 1:**
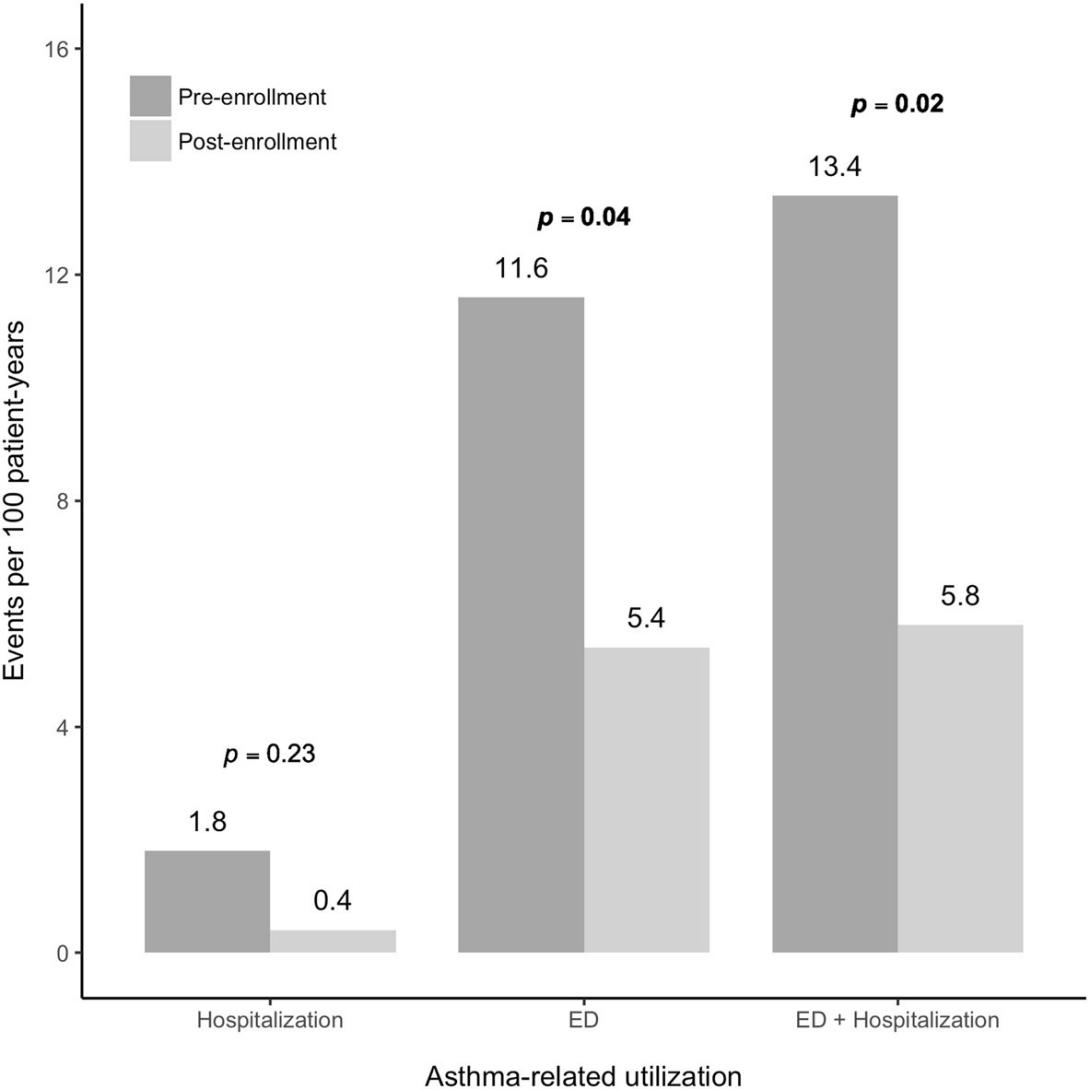
Asthma-related utilization rates pre-enrollment (dark gray) and post-enrollment (light gray) in the digital health intervention

Participants (*n* = 224) were 57% female with a mean age of 33 years (Table 1). Asthma-related healthcare utilization data are reported for the pre- and post-enrollment years (Table 2; Fig. 1). From the pre- to post-enrollment year, asthma-related hospitalizations, ED visits, and combined ED and hospitalization events declined by 1.3 (95% CI, − 0.6, 3.3, *p* = 0.23), 6.0 (95% CI, 0.9, 11.6, *p* = 0.04), and 7.6 (95% CI, 1.9, 13.3, *p* = 0.02) events per 100 patient-years, respectively. Outpatient visits per patient for asthma increased by 2.6 (95% CI, 2.2, 2.9) visits per patient-year (*p* < 0.01).

**Table 1:** Patient characteristics at baseline

Patient characteristics	*n* = 224
Mean age (SD) years	33 (23)
Age Range (years)	3–88
Female [n (%)]	128 (57)
Adults (18+) [n (%)]	132 (59)
Medication type [n (%)]	
SABA only	50 (30)
Controller only	2 (1)
Both SABA and controller	172 (69)
Controller type [n (%)]	
ICS	119 (53)
ICS/ LABA	38 (17)

**Table 2:** Pre- versus post-enrollment year rates (95% confidence intervals) in asthma-related utilization

	Pre-enrollment	Post-enrollment	Rate Difference
Hospitalizations	1.8 (0.5, 4.6)	0.4 (0.01, 2.5)	1.3 (−0.6, 3.3)
Emergency department (ED) visits	11.6 (1.6, 17.0)	5.4 (2.8, 9.4)	6.3 (0.9, 11.6)
ED + Hospitalizations	13.4 (9.0, 19.1)	5.8 (3.1, 9.2)	7.6 (1.9, 13.3)

Mean SABA use declined significantly from 0.68 puffs/day during week 1 to 0.16 puffs/day at week 52 (− 0.52 [95% CI, − 0.69, − 0.34]; *p* < 0.05). Among patients on controller medications (*n* = 76), the controller-to-total medication ratio improved during the same time points, increasing from 0.66 to 0.82 (0.16 [95% CI, 0.07, 0.25]; *p* < 0.01) (Table 3) reflecting a shift from rescue to controller use.

**Table 3:** Inhaler use improvements from week 1 to 52 following enrollment in the digital health intervention

	Week 1	Week 52	Difference	Percent change
SABA puffs/day	0.68	0.16	0.52 (95% CI: 0.34, 0.69)*	+ 76%
Controller-to-total medication ratio	0.66	0.82	−0.16 (95% CI: − 0.25, − 0.07)*	− 24%

^*^p < 0.01

## Discussion

This quality improvement project utilized a digital health intervention that provided both patients and HCPs with information about asthma medication use to inform treatment decision-making. Implementation of the program resulted in a significant decrease in asthma-related ED visits and combined ED visits and hospitalizations. The decrease in asthma-related utilization events post-intervention was consistent with the decrease in use of SABA, and was associated with a higher asthma medication ratio, a metric associated with improved asthma outcomes and lower healthcare utilization [8]. Pre-enrollment rates of asthma-related hospitalizations and ED visits were consistent with published data from commercial health plans [9]. The increase in outpatient visits noted during the study may reflect program enrollment, or greater patient and HCP awareness of asthma.

These data allow both patients and HCPs to better assess whether treatment is achieving the predetermined goals of asthma control. If treatment goals were not achieved, patients and providers could determine whether to focus on better adherence or consider other treatment options. Increased adherence has been linked to reduced high-cost utilization. In one study, every 25% improvement in adherence was associated with a 10% reduction in the risk of an asthma-related hospitalization or ED event [10].

As a single-arm quality improvement project, the results need to be cautiously interpreted because of the Hawthorne effect (i.e. confounding by regression to the mean) and potential for temporal confounding that can bias the observed estimations. Nonetheless, the correlation of reduced SABA use, improved controller-to-total medication ratio and decreased asthma-related healthcare utilization present a set of consistent findings for this quality improvement project.

In conclusion, this analysis demonstrated that digital health interventions can be incorporated into routine clinical practice, and their use may contribute to improved outcomes including reduced healthcare utilization and reduced SABA use. The information collected by the EMMs, and shared with both patients and HCPs, can promote self-management and support personalized clinical care to achieve better asthma outcomes.

## Abbreviations

ED: Emergency department
EMMs: Electronic medication monitors
FDA: Food and Drug Administration
HCP: Health care provider
HIPPA: Health Insurance Portability and Accountability Act of 1996
IRB: Institutional Review Board
LABA: Long-acting Beta Agonist
SABA: Short-acting Beta Agonist
SD: Standard deviation
